# Serum sphingolipid levels associate with upcoming virologic events and HBV genotype D in a cohort of patients with HBeAg-negative HBV infection

**DOI:** 10.1371/journal.pone.0207293

**Published:** 2018-11-15

**Authors:** Victoria Therese Mücke, Katja Jakobi, Viola Knop, Dominique Thomas, Marcus Maximilian Mücke, Kai-Henrik Peiffer, Stefan Zeuzem, Christoph Sarrazin, Josef Pfeilschifter, Georgios Grammatikos

**Affiliations:** 1 Universitätsklinikum Frankfurt, Frankfurt am Main, Germany; 2 Pharmazentrum Frankfurt, Institut für Allgemeine Pharmakologie, Frankfurt am Main, Germany; 3 Institut für Klinische Pharmakologie und Toxikologie, Frankfurt am Main, Deutschland; 4 St. Josefs-Hospital, Wiesbaden, Germany; Centre de Recherche en Cancerologie de Lyon, FRANCE

## Abstract

**Objectives:**

Sphingolipids (SLs) have been implicated as potent regulators of the hepatitis B virus (HBV) life cycle. We investigated the SL biomarker potential regarding virologic endpoints in a prospective subgroup of patients with HBeAg-negative chronic HBV infection.

**Methods:**

From 2009–2016 98 patients with HBeAg-negative HBV infection were prospectively followed over four years. Clinical, laboratory and imaging data were evaluated annually. SLs were assessed in available serum probes via liquid chromatography coupled to tandem mass spectrometry.

**Results:**

Of those 98 patients, 10 (10.2%) showed HBV reactivation, 13 (13.2%) lost HBsAg and 9 (9.1%) gained status of HBsAg-/HBsAb-coexistence, whereas 66 (67.3%) had no events. Within the four-year analysis sphingosine (p = 0.020), sphinganine (p<0.001), dhS1P (p<0.001), C16DHC (p<0.01) and C20Cer (p<0.001) showed a significant upregulation in patients without virologic events, C18Cer significantly decreased (p<0.001). At baseline decreased S1P-, dhS1P- and C16Cer-levels were observed in patients with upcoming status of HBsAg-/HBsAb-coexistence. S1P and dhS1P levels were elevated HBV genotype D infected patients.

**Conclusions:**

In a prospective cohort of patients with a HBeAg-negative HBV infection, serum SLs associated with the virologic course and HBV genotype D. Further studies are required to elucidate SLs as potential novel predictors of the course of HBeAg-negative HBV infection.

## Introduction

Approximately 350 million people worldwide are chronically infected with the hepatitis B virus (HBV) [1]. HBV is ranked as the tenth leading cause of death [2] as it accounts for 30% of liver cirrhosis cases and is attributed as the leading cause to 53% of hepatocellular carcinoma (HCC) diagnosed [3]. In the clinical setting most patients with chronic HBV infection present with positive hepatitis B surface antigen (HBsAg), negative hepatitis B envelope antigen (HBeAg), normal aminotransferases and low viral load (HBV DNA <2000 IU/ml) [4], former called “inactive carrier”, and thus considered as not eligible for antiviral treatment [5]. In HBsAg-positive/HBeAg-negative patients, with low HBV-DNA, the risk of liver related morbidity and mortality is reported to be low and strongly influenced by cofactors [6, 7]. Nevertheless, most publications on this topic refer to European cohort studies, thus knowledge on natural history data in non-genotype D dominant regions of HBV infection (i.e. Asia, Africa and South America) may vary. The associated risks of different HBV genotypes concerning severity of liver disease and HCC development however are a controversial issue [8, 9]. Current surveillance intervals and indication for therapy is mostly driven by HBV DNA levels, liver inflammation and occurrence of liver fibrosis [5]. In the case of treatment indication the currently available antiviral drugs rarely achieve HBV eradication and little is known about long-term side effects or long-term antiviral resistance [10]. Due to the lack of reliable early predictors of HBV reactivation, induced liver fibrosis and early oncogenesis, new biomarkers are pivotal to ensure sufficient risk stratification in every HBV infected individual, especially in those not eligible for immediate antiviral treatment.

**Fig 1:**
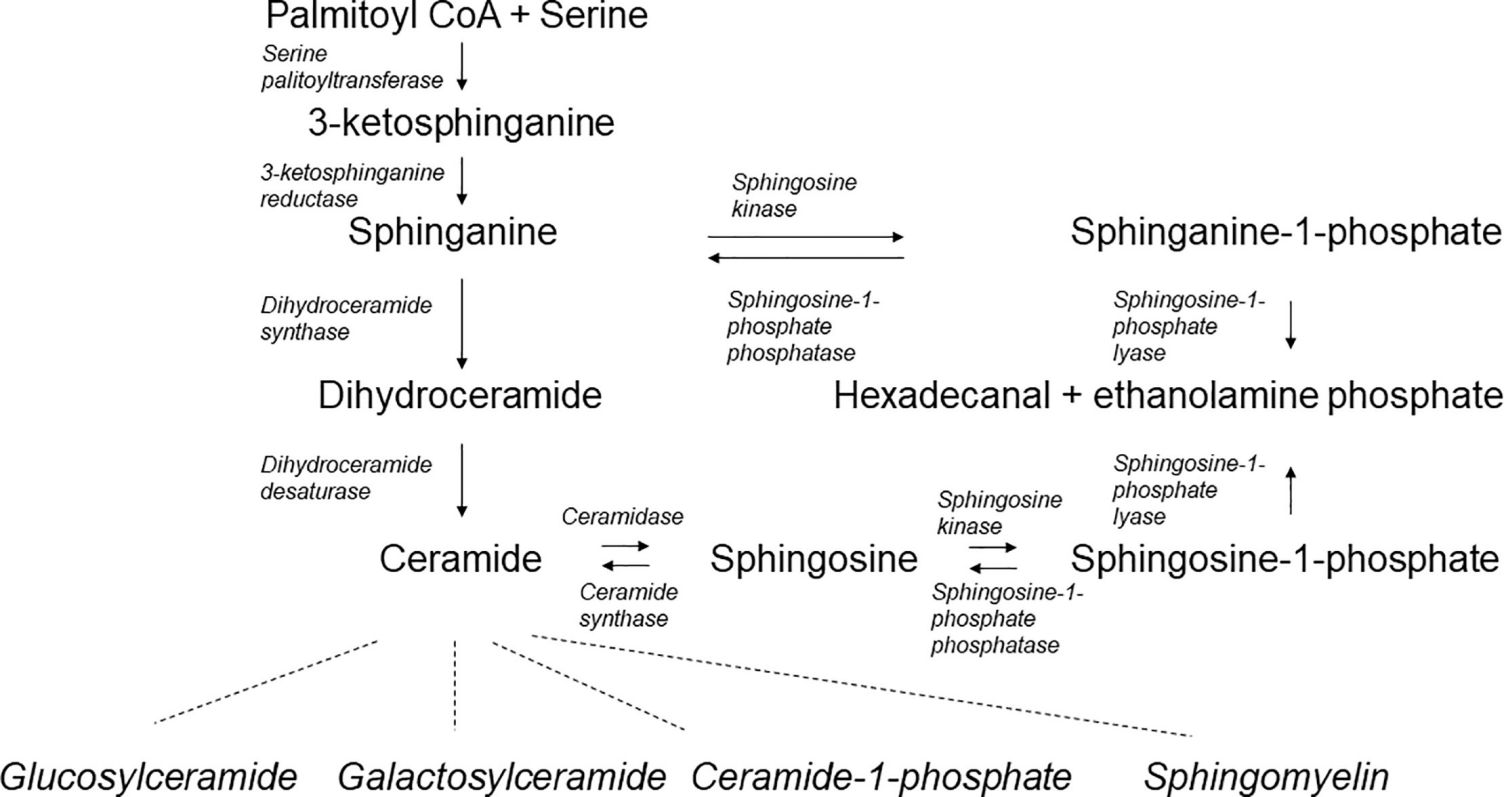
Overview of core sphingolipid pathways. The sphingolipids analysed in our study are highly interconnected and play central roles in sphingolipid metabolism.

Sphingolipids (SLs) are complex bioactive molecules which play an important role in the pathophysiology and pathogenesis of multiple diseases due to their substantial implication in cellular homeostasis. As in recent reviews well summarized, SL metabolic pathways are highly interconnected [11, 12] (Fig 1). SLs regulate signalling and especially the balance between apoptosis and proliferation [13]. The important roles of SL metabolites, especially ceramide and sphingosine-1-phosphate (S1P), in the pathophysiology of oncogenesis have been well established [14]. Ceramide, known for its pro-apoptotic effects, has been recently in focus regarding new HCC treatment [15]. Using combination therapies of sorafenib plus recombinant human acid sphingomyelinase, which hydrolyses sphingomyelin to ceramide, on Huh7 xenografts, a synergistic effect on reducing HCC tumour volume and blood vessel density could be demonstrated. Recently our group showed significant upregulations of serum C16-ceramide and serum S1P in patients with liver cirrhosis and HCC compared to cirrhotic patients without HCC [16]. Moreover, we identified SL profiles to predict fibrosis progression and sustained viral response rates in patients with chronic hepatitis C virus (HCV) infection [17], as well as promote HCV persistence upon acute infection [18]. In the HBV infected subgroup, we were not able to see alternating SL levels concerning fibrosis progression [17]. Yet, we could identify significant differences in SL profiles between HCV and HBV infected patients. Studies on Asian cohorts reported SL correlations regarding the degree of hepatic injury in chronic HBV infection [19, 20]. Furthermore, current studies revealed a key role of SLs in the pathophysiology of viral infections and identified SLs as promising new therapeutic target points for the inhibition of hepatotropic virus replication [21, 22]. Most recently, Sanada et al. showed that ceramide-triggered extracellular vesicles are even capable of transmitting HBV DNA into hepatocytes during HBV infection, which would imply a new alternative route of HBV infection [23]. Considering the mechanistic roles and former clinical observations in HBV cohorts we hypothesized that SL metabolites may play a pivotal role in the HBV life cycle. Consequently, we aimed to analyse longitudinally the serologic SL profiles in a European multicentre cohort of patients with HBsAg-positive/HBeAg-negative HBV infection focusing on upcoming virologic events, HBV-genotype differences and fibrosis progression.

## Patients and methods

### Patients’ selection

In this multicentre trial, we characterized SL profiles of patients with chronic HBeAg-negative HBV infection. Between June 2009 and October 2016 serum samples of HBV patients included in the ongoing prospective HBV longitudinal study (ALBATROS, NCT01090531) were routinely stored. This great multicentre prospective study includes twelve participating centres (S1 Table) and comprises more than 300 patients, mono-infected with HBV (HBsAg-positive and HBV DNA positive for > 6 months) and not considered for antiviral treatment at the time of study inclusion (HBeAg negative, ALT values ≤ 2x ULN, viral load < 100.000 IU/mL). Baseline and follow up characteristics, laboratory tests and fibrosis stage were assessed equally in all participating centres. Blood samples were taken on fasting patients and liver fibrosis was assessed using transient elastography (TE) by *FibroScan* at the same time. In this study, we investigated all available serum samples of patients who had either a virologic event (start on antiviral treatment due to HBV reactivation [disease progression / increasing viral load / fibrosis progression / significant elevation of transaminases], loss of HBsAg, gaining status of HBsAg-positive and HBsAb-positive) or at least three to four annually follow-up visits, all meeting the following criteria: Age 18–79, HBV treatment-naïve, BMI < 35, documented TE at baseline, genotypes A or D and B, C and E only with virologic event (S1 Fig). The clinical and trial database provided further demographic and clinical characteristics including age, sex, demographic characteristics, BMI, routine and virologic laboratory values.

### Quantification of HBV DNA and determination of sphingolipid concentrations by high-performance liquid chromatography tandem mass spectrometry

HBV DNA was tested in plasma samples using COBAS AmpliPrep/COBAS TaqMan HBV DNA assay, version 2.0 (CAP/CTM HBV; Roche Diagnostics). According to manufacturer’s information the lower limit of quantification of the CAP/CTM HBV assay is 20 IU/mL. High-performance liquid chromatography tandem mass spectrometry was performed per protocol as recently described [[24]].

### Statistical analysis

Statistical calculations were performed by using BiAS software for Windows (version 11.05; Epsilon-Verlag, Darmstadt, Germany). Analysis for the presented box plots was performed with GraphPad Prism for Windows (v5.02; GraphPad Software Inc., San Diego, CA). Longitudinal non-parametric calculations in patients’ parameters were performed using Wilcoxon-matched pairs analyses, two-groups non-parametric calculations were done by Mann-Whitney-U test, multiple groups non-parametric calculations were performed by Kruskall-Wallis test. Rank correlations were calculated using Spearman and Kendall method. P-values <0.05 were considered statistically significant.

### Ethical approval

The study was performed in accordance with the Declaration of Helsinki. All patients had signed a written informed consent from the original multicentre ALBATROS-trial (NCT01090531) before study inclusion. It included the approval for the asservation of serum samples for future analyses. The focus on SL parameters was additionally approved by the ethics committee of the principal trial centre the Ethikkommission des Fachbereichs Medizin of the Johann Wolfgang Goethe-Universität Frankfurt (file no. 4/09 with project no. SGI.05-2009). Standards of good clinical practice were followed during patients care and study conduct at all times.

## Results

### Patients’ characteristics

According to the above described criteria 98 patients with chronic, HBeAg-negative, inactive chronic HBV infection and not considered for antiviral therapy were included. Among these 64 (65%) were infected by genotype D, 15 (15%) by genotype A, 2 (2%) by each genotype B and C and 1 patient (1%) was infected by genotype E. In 14 patients (14%) no genotype was determinable due to non-quantifiable viral load. Table 1 depicts detailed baseline characteristics of the included patients. During the four-year follow-up period, almost 33% (n = 32) of this patients’ subgroup were documented to have one of the following virologic events: (i) start on antiviral therapy due to HBV reactivation (n = 10), (ii) loss of HBsAg (n = 13), (iii) gaining simultaneous status of HBsAg- and HBsAb-positivity (n = 9). In sixty-seven percent (n = 66) no virologic events occurred. We did not observe any acute (on chronic) liver failure, significant progression of fibrosis in yearly TE measurements (S2 Fig), formation of hepatocellular carcinoma (HCC) or deaths in our cohort of inactive HBV carriers.

**Table 1:** Patients' characteristics at baseline.

Parameters	Patients n = 98
Age (years), median (range)	40.5 (18–67)
**Gender**	
Female, n (%)	55 (56)
Male, n (%)	43 (44)
**Ethnicity**	
Caucasian, n (%)	45 (46)
Oriental, n (%)	29 (30)
Black, n (%)	9 (9)
Asian, n (%)	9 (9)
Not applicable, n (%)	6 (6)
Body mass index kg/m^2^: median (range)	25 (18.-33.8)
Transient elastography (kPa): median (range)	5.3 (2.5–12)
**Hepatitis B virus genotype**	
A, n (%)	15 (15)
B, n (%)	2 (2)
C, n (%)	2 (2)
D, n (%)	64 (65)
E, n (%)	14 (14)
**Biochemical parameters**	
ALT (IU/L), median (range)	25.5 (11–98)
AST (IU/L), median (range)	26 (8–119)
GGT (IU/L), median (range)	18 (3–172)
Bilirubin (mg/dl), median (range)	0.5 (0.1–1.7)
Creatinine (mg/dl), median (range)	0.76 (0.47–1.52)
Chol (mg/dl), median (range)	188 (106–282)
TG (mg/dl), median (range)	87 (44–613)
LDL (mg/dl), median (range)	106 (50–191)
HDL (mg/dl), median (range)	57.5 (22–114)
HBV viral load (IU/ml), median (range)	632.5 (0–97000)
HBsAg (IU/ml), median (range)	1346 (0.1–31940)
**Virologic events**	
No virologic events, n (%)	66 (67)
HBsAg Loss, n (%)	13 (13)
Reactivation, n (%)	10 (10)
HBsAg plus HBsAb-status, n (%)	9 (9)

Median with range or number of patients in percent in brackets. Abbreviations: ALT, alanine transferase; AST, aspartate transferase; GGT, gamma glutamyl transferase; Chol, cholesterol; TG, triglyceride; LDL, low density lipoprotein; HDL, high density lipoprotein; HBV, hepatitis B virus; HBsAg, hepatitis B virus surface antigen; HBsAb, hepatitis B virus surface antibody. Missing data: Bilirubin level is missing in 1 patient, cholesterol and triglyceride levels are missing in 2 patients, HDL levels are missing in 10 patients, LDL levels are missing in 11 patients, HBsAg levels are missing in 27 cases.

### Longitudinal analyses reveal decreasing serum sphingolipids in concordance to viral and HBsAg loads in patients without upcoming virologic events

**Fig 2:**
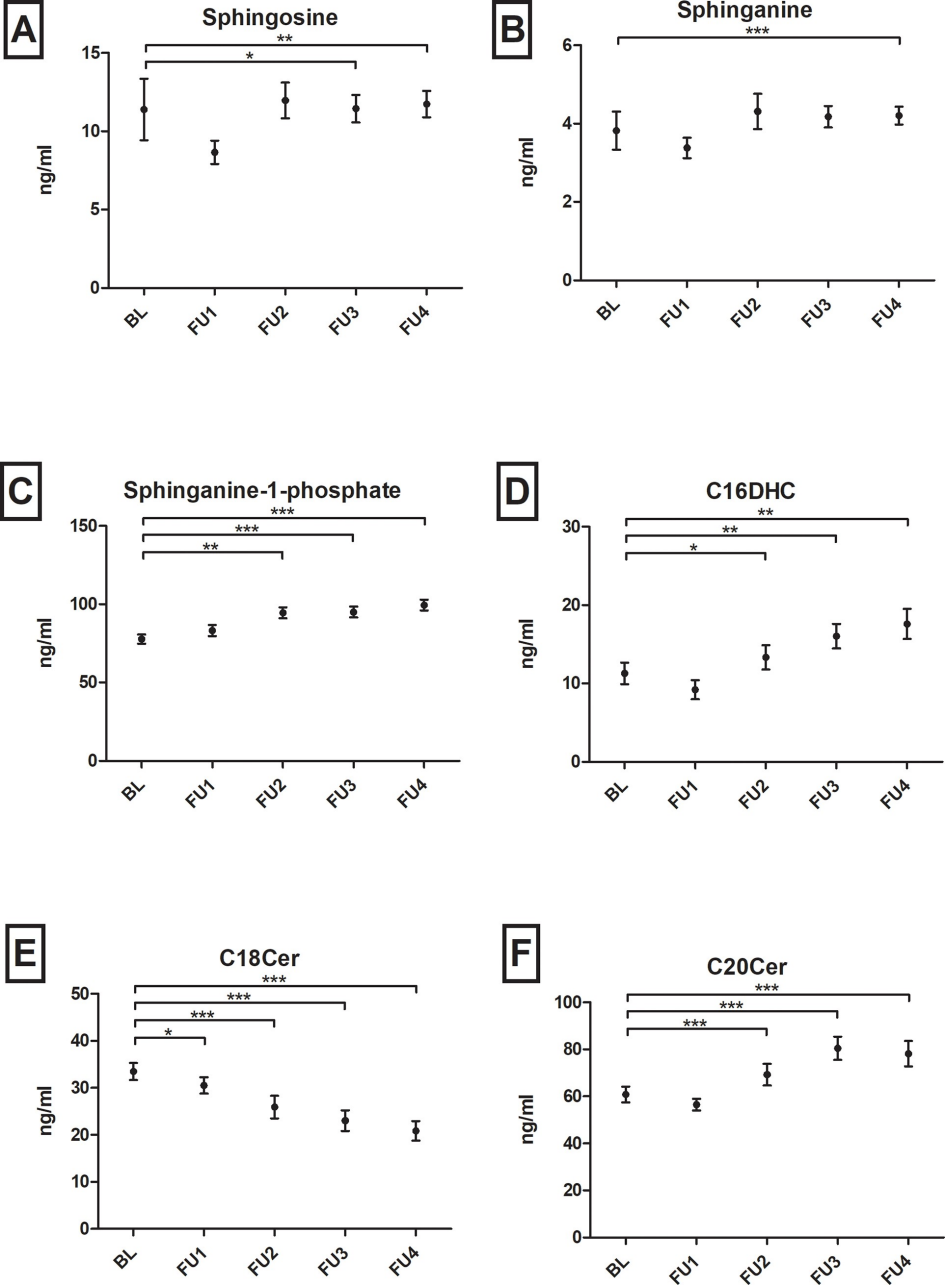
**Course of (A) sphingosine, (B) sphinganine, (C) sphinganine-1-phosphate, (D) C16-dihydroceramide (C16DHC), (E) C18- and (F) C20-ceramide (C18/C20Cer) in patients with no virologic events from baseline (BL) over a follow-up (FU) period of four years (1–4).** While C18Cer levels decrease, all other sphingolipid parameters are continuously increasing over time. Statistically significant differences are indicated by asterisks. "*"p<0.05, "**"p<0.01, "***"p<0.001. Bars depict mean +/- standard mean error.

**Fig 3:**
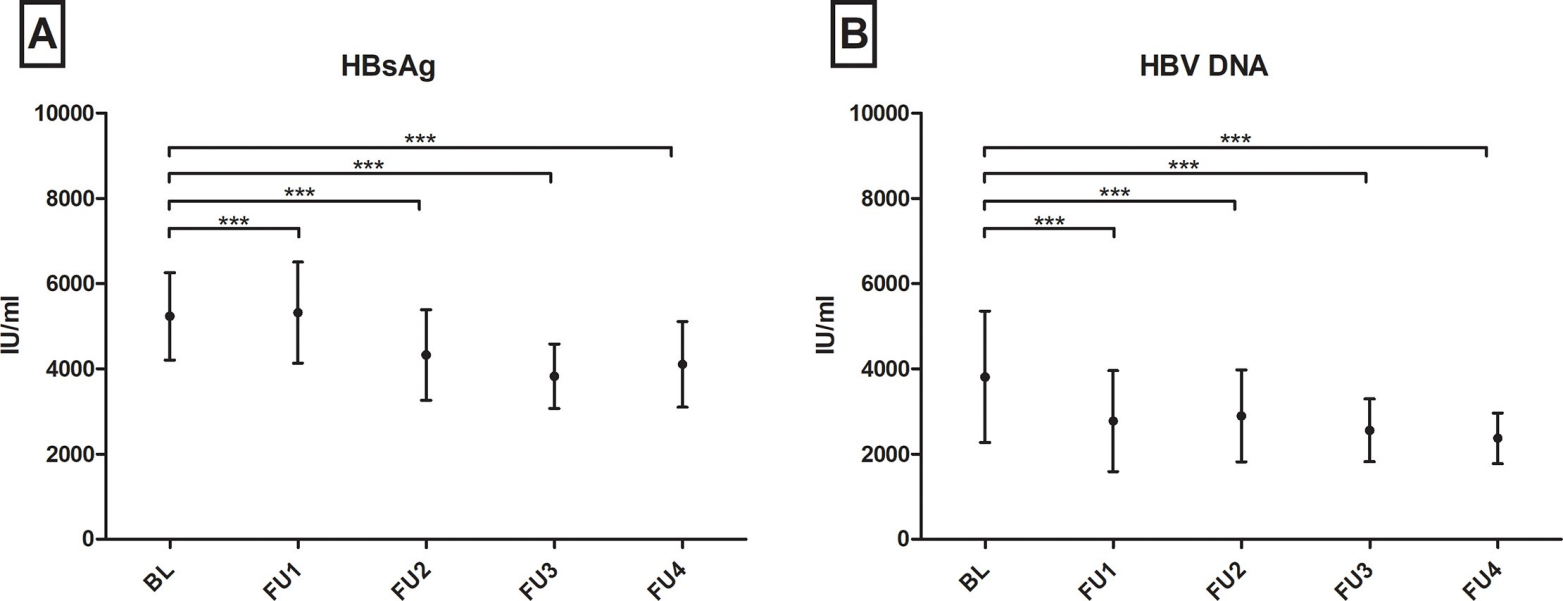
Course of hepatis B virus surface antigen (HbsAg) and hepatitis B virus (HBV) deoxyribonucleic acid (DNA) in patients with no virologic events from baseline (BL) over a follow-up (FU) period of four years (1–4). Both parameters decrease significantly over time. Statistically significant differences are indicated by asterisks. "*"p<0.05, "**"p<0.01, "***"p<0.001. Bars depict mean +/- standard mean error.

As depicted in Fig 2 specific SL profiles changed significantly in patients with chronic HBV infection without virologic events over time. Over a period of four years, levels of sphingosine (p = 0.020), sphinganine (p<0.001), dhS1P (p<0.001), C16DHC (p<0.01) and C20Cer (p<0.001) continuously increased, whereas C18Cer levels decreased (p<0.001) as compared to baseline. S3 Fig visualizes remaining SL parameters without significant longitudinal changes. In parallel, HBV viral load and HBsAg levels decreased significantly in longitudinal analyses (Fig 3). Direct correlations of the respective delta-values (BL minus FU4) of HBV viral load/HBsAg levels and the depicted SL parameters were not significant. In other patients’ subgroups with virologic events these tendencies could not be observed. Wilcoxon-matched pairs tests analysing SL parameters in the last visit before and the first visit after HBsAg-loss did not show significant differences in SL metabolites (n = 13, p>0.05).

### Baseline serum S1P-, dhS1P- and C16Cer-levels are down-regulated in patients with upcoming HBsAg/HBsAb-positivity

**Fig 4:**
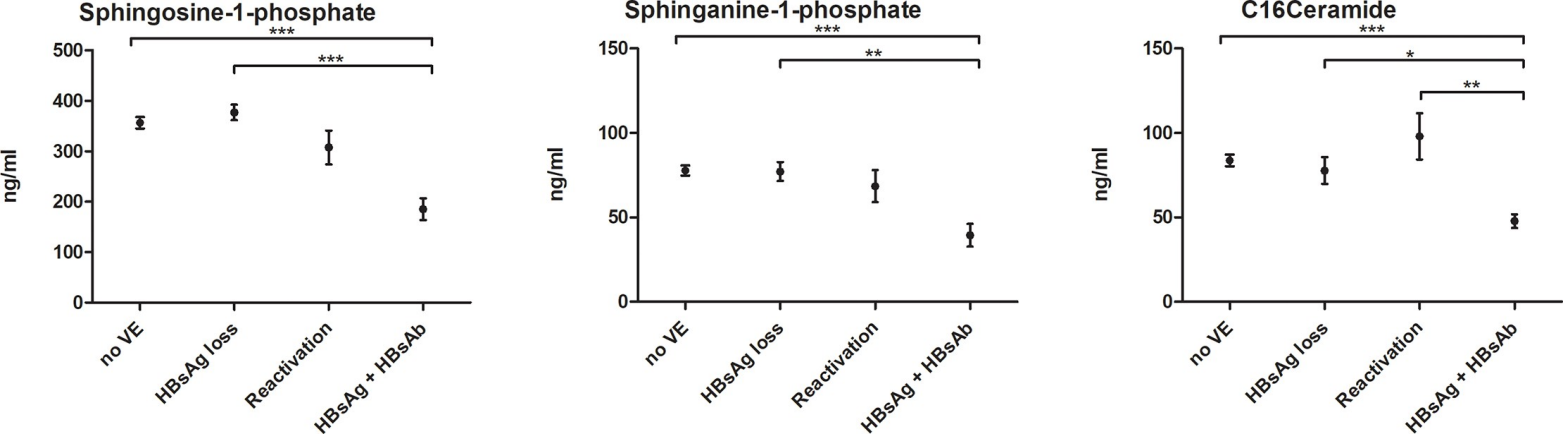
Kruskall-Wallis analyses at baseline between patients with different upcoming virologic events. Future HBsAg plus HBsAb status is associated with significant low alterations in serum sphingosine-1-phospahte, sphinganine-1-phosphate and C16Ceramide. already at date of study inclusion. Abbreviations: VE, virologic event; HBsAG, hepatitis B virus surface antigen; HBsAb, hepatitis B virus surface antibody. Bars depict mean +/- standard mean error.

Serum concentrations of various SL metabolites were compared between patients with and patients without virologic events. In sub-group analyses (patients without upcoming virologic event, patients with upcoming HBsAg loss, patients with upcoming HBV reactivation and patients with upcoming HBsAg-/HBsAb-positive status), S1P, dhS1P and C16Cer were significantly down-regulated in patients with upcoming simultaneous HBsAg- and HBsAb-positivity (Fig 4). These differences were greatest in comparison to no virologic event (p<0.001) and HBsAg loss (p<0.05). Non-significant differences in further SL parameters are visualized in supporting Fig 4. Multivariate analyses regarding HBsAg/HBsAb-positivity still revealed S1P (Odds-Ratio [OR] = 0.977; 95%-confidence interval [CI] = 0.961–0.994; p = 0.007) and C16Cer (OR = 0.911; CI = 0.847–0.980; p = 0.012) as independent factors (Table 2). In addition, HBV DNA levels at baseline were significantly lower in patients with upcoming HBsAg loss (p = 0.005). Of note, AST, ALT, GGT, TG, HDL, LDL and Chol could not predict any upcoming virologic events (p>0.05).

**Table 2:** Multivariate analyses for HBsAg-/HBsAb-positivity.

	*Univariate Analysis*		*Multivariate Analysis*	
***Variable***	***P* value**	**OR (95% CI)**	***P* value**	**OR (95% CI)**
*Age*	0.788	1.009 (0.948–1.073)		
*Gender*	0.201	2.917 (0.565–15.063)		
*Body mass index*	0.823	0.980 (0.817–1.175)		
*ALT*	0.558	0.984 (0.933–1.038)		
*AST*	0.202	0.928 (0.828–1.041)		
*Bilirubin*	0.143	0.069 (0.002–2.455)		
*Sphingosine-1-phosphate*	0.0004	0.977 (1.964–0.990)	0.007	0.977 (0.961–0.994)
*Sphinganine-1-phosphate*	0.0004	0.929 (0.893–0.968)		
*C16-ceramide*	0.0008	0.898 (0.843–0.957)	0.012	0.911 (0.847–0.980)

Abbreviations: OR, odds-ratio; CI, confidence interval; ALT, alanine transferase; AST, aspartate transferase.

### S1P- and dhS1P-levels are up-regulated in patients with HBV genotype D

**Fig 5:**
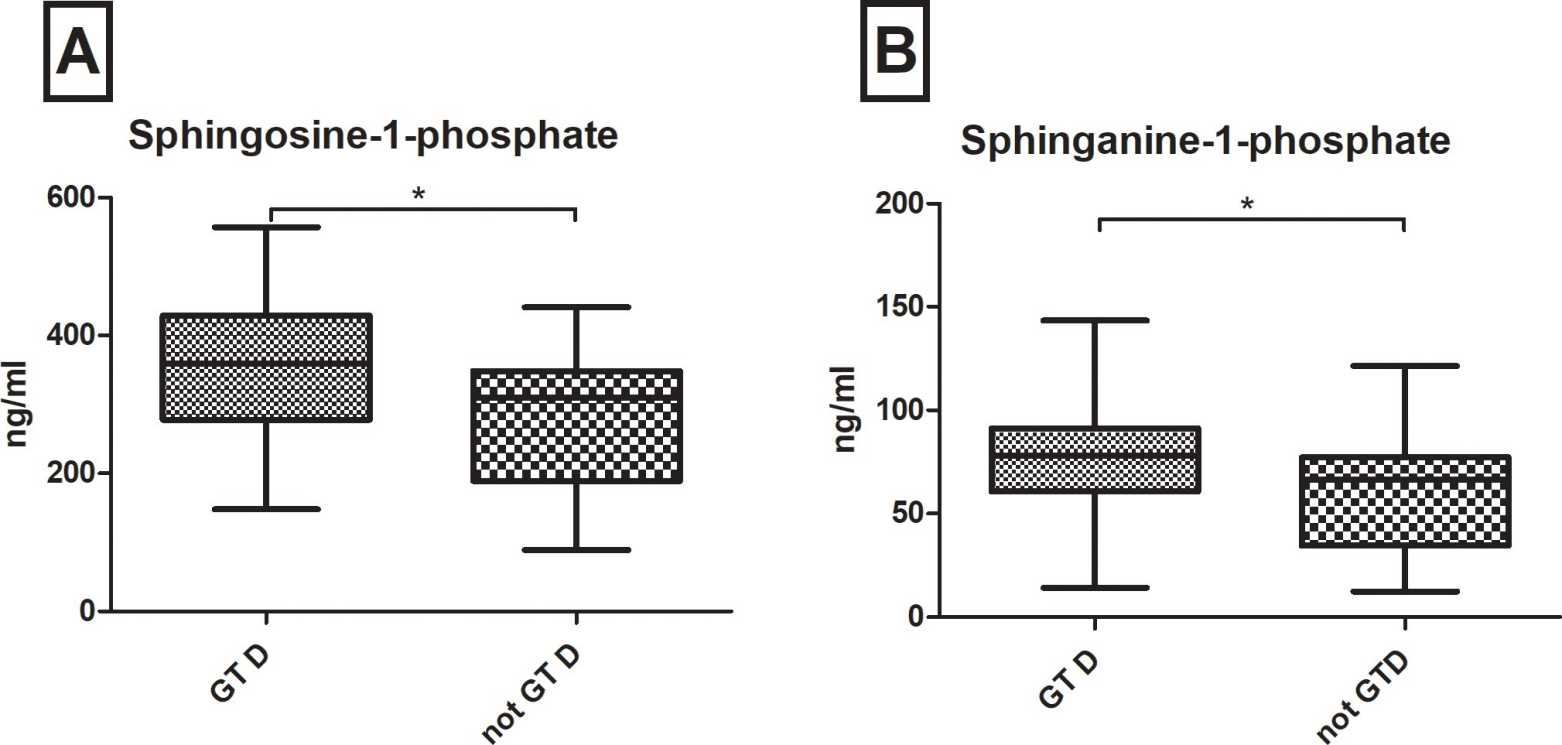
**At baseline, serum sphingosine-1-phosphate (A) and sphinganine-1-phosphate (B) are up-regulated in patients infected with hepatitis B virus genotype D (GT D) compared to other genotypes (not GT D).** Statistically significant differences are indicated by asterisks: "*" = p<0.05.

Additionally, S1P (p = 0.012) and dhS1P (p = 0.043) were up-regulated in patients with genotype D compared to all other HBV genotypes (Fig 5). In a multivariate logistic regression model including further variables of age, gender, BMI, transaminases and lipids, HDL (OR = 0.961; CI = 0.927–0.998; p = 0.019) and especially S1P (OR = 1.008; CI = 1.002–1.015; p = 0.003) were independently associated with HBV genotype D (Table 3).

**Table 3:** Multivariate analyses for hepatitis B virus genotype D.

	*Univariate Analysis*		*Multivariate Analysis*	
***Variable***	***P* value**	**OR (95% CI)**	***P* value**	**OR (95% CI)**
*Age*	0.108	0.960 (0.912–1.009)		
*Gender*	0.154	0.405 (0.117–1.405)		
*Body mass index*	0.322	1.077 (0.930–1.246)		
*ALT*	0.951	1.001 (0.968–1.035)		
*AST*	0.748	1.007 (0.965–1.051)		
*Sphingosine-1-phosphate*	0.003	1.009 (1.003–1.015)	0.008	1.008 (1.002–1.015)
*Sphinganine-1-phosphate*	0.017	1.031 (1.007–1.057)		
*Cholesterol*	0.546	0.996 (0.981–1.010)		
*Triglyceride*	0.408	1.004 (0.994–1.015)		
*High-density lipoprotein*	0.014	0.956 (0.923–0.991)	0.037	0.961 (0.927–0.998)
*HBV viral load*	0.870	1.000 (1.000–1.000)		

Abbreviations: OR, odds-ratio; CI, confidence interval; ALT, alanine transferase; AST, aspartate transferase. HBV, hepatitis B virus. Missing data: cholesterol and triglyceride levels were missing in 2 patients; HDL levels were missing in 10 patients.

### Correlations of sphingolipids with demographic and biochemical patient data

By using Spearman’s rank correlations, we identified associations between serum SL levels between demographic and biochemical patient data. Significant baseline correlations were seen between nearly all serum ceramide (Cer) and dihydroceramide (DHC) parameters and cholesterol (Chol) levels and some triglyceride (TG) levels. Some SL parameters correlated with age (sphingosine, r = 0.228, p<0.05; sphinganine, r = 0.267, p<0.05; Cer24 r = 330, p<0.001; C24DHC, r = 0.239, p<0.05 and C24:1DHC, r = 0.226, p<0.05) and BMI (sphingosine, r = 0.234, p<0.05; sphinganine, r = 0.218, p<0.05; C24:1Cer, r = 0.211, p<0.05). No baseline correlations were found between serum SL parameters and transaminases, HBV viral load, alpha-fetoprotein (AFP) or stage of fibrosis determined by TE (Table 4).

**Table 4:** Correlations of serum sphingolipids of all patients (n = 98) with age, body mass index (BMI), aspartate transferase (AST), alanine transferase (ALT), cholesterol (Chol), triglycerides (TG), transient elastography (TE), alpha-fetoprotein (AFP), viral load (VL) and hepatitis B virus surface antigen (HBsAg).

*SL*	*Age*	*BMI*	*AST*	*ALT*	*Chol*	*TG*	*TE*	*AFP*	*VL*	*HBsAg*
*Sphingosine*	0.228*	0.234*	-0.035	0.077	0.014	0.119	0.071	0.172	-0.005	-0.064
*Sphinganine*	0.267*	0.218*	0.013	0.120	0.111	0.146	0.014	0.070	-0.013	-0.034
*Sphingosine-1-phosphate*	-0.129	-0.048	0.158	0.115	-0.040	-0.040	0.070	0.194	-0.063	-0.059
*Sphinganine-1-phosphate*	-0.108	-0.027	0.155	0.053	-0.097	-0.025	-0.008	0.188	0.034	-0.113
*C24Cer*	0.330***	0.120	0.110	0.103	0,667***	0.339***	-0.036	0.075	-0.065	-0.070
*C16Cer*	0.034	-0.153	0.004	-0.013	0.416***	0.070	-0.017	0.020	0.101	-0.035
*C14Cer*	0.064	-0.100	-0.061	-0.094	0.159	0.052	-0.038	0.009	0.098	0.159
*C18Cer*	0.160	-0.011	0.105	0.064	0.423***	0.173	0.112	-0.005	0.007	-0.034
*C20Cer*	0.094	0.027	0.103	0.168	0.290**	0.292**	0.044	0.031	-0.131	-0.043
*C24:1Cer*	0.195	0.034	0.170	0.068	0.518***	0.188	0.006	0.129	-0.022	-0.041
*C16DHC*	-0.046	-0.162	-0.004	0.036	0.056	0.100	-0.038	0.059	0.045	0.243
*C18DHC*	0.196	0.216	0.191	0.037	0.413***	0.218	-0-018	0.036	-0.143	-0.181
*C24DHC*	0.239*	0.197*	0.087	0.146	0.603***	0.260*	0.059	0.114	-0.063	-0.051
*C24:1DHC*	0.226*	0.211*	0.151	0.140	0.433***	0.240*	0.106	0.113	-0.066	-0.211

Correlations are evaluated by Spearman’s rank correlation coefficient rho (r). Significant correlations are shown in bold and are indicated in the corresponding figures:

*p<0.05

**p<0.01

***p<0.001. Missing data: cholesterol and triglyceride levels were missing in 2 patients, AFP levels were missing in 3 patients, HBsAg levels are missing in 27 cases.

### C14Cer and C16Cer are increased after HBV reactivation

**Fig 6:**
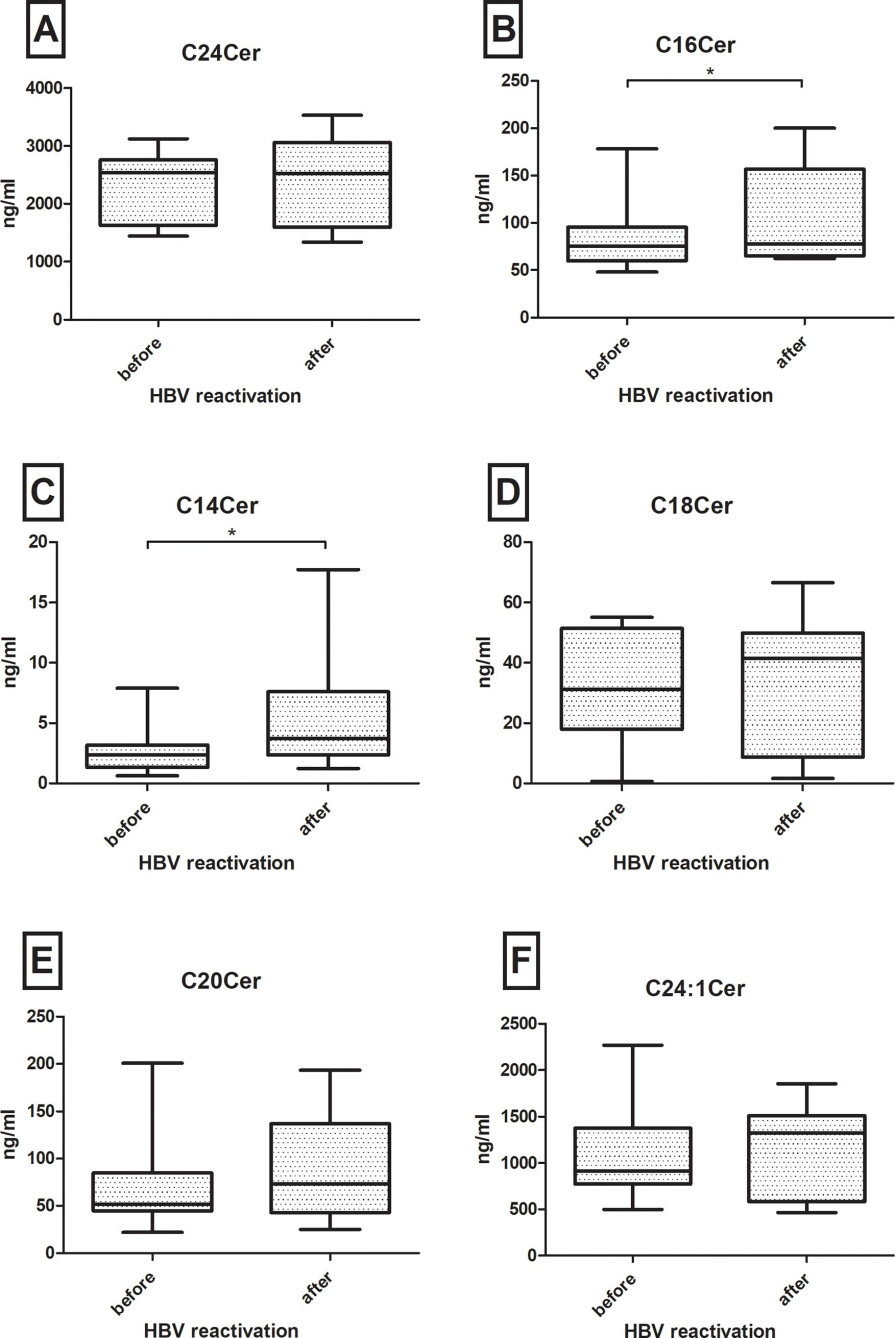
**Alterations of (A) C24Cer, (B) C16Cer, (C) C14Cer, (D)C18Cer, (E) C20Cer and (F) C24:1Cer in all patients with hepatitis B (HBV) reactivation.** Sphingolipids are compared at last visit before and next visit after HBV reactivation. There are no significant dynamics in long chain ceramides C18Cer and greater (A, D-F), but significant increase of C16Cer (B) and C14Cer (C). Statistically significant differences are indicated by asterisks. "*"p<0.05, "**"p<0.01, "***"p<0.001. Bars depict mean +/- standard mean error.

We further analysed SL metabolites of the named ten patients who suffered from HBV reactivation during the trial. Using Wilcoxon-matched-pair tests we compared SL concentrations the last visit before HBV reactivation with SL concentration the first visit after HBV reactivation. We could only observe significant SL changes in C14Cer and C16Cer (all ceramides are visualized in Fig 6). But in tendency, most SL parameters showed increased concentrations after HBV reactivation. Further SL parameters are depicted in supporting Figs 5 and 6.

## Discussion

According to an increasing number of reports on the functional role of SLs in liver biology and pathophysiology [25], in the present study we aimed to investigate serologic SL variations in a subgroup of a large longitudinal European multi-centre cohort of patients with HBeAg-negative HBV infection, who were not intended to receive antiviral therapy according to current European guidelines [5]. The challenge in patients with HBeAg-negative HBV infection is to not miss first signs of HBV complications, such as smouldering inflammation, development of HCC without cirrhosis and slightly progressing fibrosis as well as to outbalance advantages, need and side-effects of antiviral therapy. Furthermore, adequate follow-up intervals for these asymptomatic patients are still inadequately defined. As we have already identified in recently published own studies, serum SL’s show a predictive potential regarding fibrosis progression and sustained viral response rates under PEG-IFN–treatment in HCV [17]. Thereby, we observed significant variations of the serologic SL profile among HCV- and HBV-patients. Additionally, as already shown by others, SLs are involved in HBV replication and HBV infection pathways: Tatematsu et al. already declared myriocin -a specific serine palmitoyl transferase (SPT) inhibitor- which constitutes the key enzyme for the initiation of de-novo ceramide synthesis, as a possible novel target for new antiviral HBV therapies, referring to the synergistic suppression of HBV replication in in vivo models [22]. As mentioned above, HBV DNA containing extracellular vesicles are primarily able to infect new hepatocytes, resistant to antibody neutralization, leading to the question of evolving new strategies to inhibit these relevant immune escaping infectious pathways [23]. Thus, in the present study we focused on SLs as potential biomarkers regarding HBV virologic events, such as loss of HBsAg or starting on antiviral therapy due to HBV reactivation.

Interestingly, SL levels significantly varied over time in patients with chronic HBV infection without upcoming virologic events (Fig 2). These variations were in concordance with decreasing HBV viral load and HBsAg levels over the same time (Fig 3). Therefore, this longitudinal association may show the potential of SLs to reflect HBV replication activity in serum probes. These SL tendencies could not be observed in patients with loss of HBsAg or in patients with upcoming HBV reactivation. Instead SL metabolites mainly increased after HBV reactivation, e.g. significantly C14Cer and C16Cer. This is likely reflecting increased cell-turn over and pro-proliferative actions. Limited by the small group of only ten patients, including four who already showed HBV reactivation at visit FU1, unfortunately we cannot sufficiently analyse changes in SL metabolites before HBV reactivation e.g. in comparison to baseline parameters. Furthermore, in our analyses S1P, dhS1P and C16Cer baseline levels were significantly associated with the upcoming virologic event of HBsAg- and HBsAb-positivity, predictive over the next four years of observation. This status of HBsAg- and HBsAb-coexistence is still a subject of debate, but different authors state it as a marker for possible immune selection and change of clinical courses. Liu et al. stated that in a Chinese HBV population it may be associated with a higher frequency of mutations in the alpha determinant of HBV genotype C [26, 27] and Seo et. al. also postulated this coexistence as potential risk factor for HCC [28]. Other authors identified HBsAg- and HBsAb-coexistence in 4.9% of their Asian cohorts, but not leading to a selecting of HBV escape mutants [29]. In our European cohort median HBsAg- and HBsAb-levels and HBV viral load in patients with coexistence were low and decreased over time. It is assumable, that these patients may undergo a very slow seroconversion. In line with our longitudinal observations, the decreased S1P-, dhS1P- and C16Cer-levels may reflect again low HBV replication and upcoming seroconversion. Limited by the small number of patients achieving virologic events, no significant differences could be seen in other smaller subgroup analyses.

HBV genotypes have been associated to different, sometimes controversial clinical and virologic courses of HBV infection [30, 31]. Some studies revealed that HBV genotypes A and B are associated to a better virologic response to IFN-therapies than genotypes C and D [32, 33]. While others showed that compared to genotype A and C, genotype D and B are associated with worse clinical and virologic outcome [34, 35]. A meta-analysis of Wang et al. calculated a higher risk for HCC development for patients infected by HBV genotype C than A, B or D [36]. However, further studies revealed that development of liver cirrhosis and HCC were also associated to specific gene mutations especially in regions that regulated transcription, frequently observed in patients with genotype D [37]. For patients’ individual risk stratification, many authors see benefits genotyping HBV infected patients [9, 38]. In our European cohort genotype D and A were predominantly represented in concordance with epidemiologic studies [39]. By comparing our largest genotype subgroup (genotype D) with the other genotypes, we could interestingly observe significant elevated serum levels of S1P and dhS1P. Zeng et al. stated that S1P induces epithelial-mesenchymal transition of hepatocellular carcinoma [40] and inhibits cell apoptosis via syndecan-1 [41]. Furthermore, Li et al. and Yang et al. observed that S1P and S1P-receptors are up-regulated in liver fibrosis and liver fibrosis related angiogenesis [42, 43]. In line with these studies, our observed results may also reflect a more pro-proliferative hepatic metabolism in patients with HBV genotype D, including increased risk for liver fibrosis and HCC.

We state some limitations in our study. First, we depended on relatively small subgroups with virologic events, mainly in concordance with epidemiologic phenomena. Larger patients’ cohorts and subgroup analyses need to confirm our findings. Second, due to very low viral loads in patients’ probes, HBV deep sequencing was not performed, while cofactors such as precore mutations, core promoter mutations or quasi species are likely to influence virologic course and HBsAg-/HBsAb-coexistence. These may relate to SL profiles. Third, paucity of proven pathophysiological pathways makes it difficult to explain our results in specific mechanistic roles in HBV infected humans. Confronted by the great complexity of SL metabolism and HBV life cycle further in vivo and in vitro studies are needed.

Nevertheless, in contrast to Zheng’s et al. HBV collective [19] our cohort did not undergo significant different progressing stages of chronic HBV infection, such as acute (on chronic) liver failure, HCC or different stages of liver fibrosis and cirrhosis. Thus, we analysed serologic SL concentrations in “steady state” HBV-infected patients avoiding biased results by hepatic decompensation, progressing liver fibrosis or HCC occurrence. Our results suggest serum SL parameters to have predictive potential in chronic HBeAg–negative HBV infected patients. Conclusory, to our knowledge, we were the first to identify serum SL parameters as possible predictive parameters in a prospective European cohort of patients with inactive HBeAg-negative HBV infection. Serum sphingosine (p = 0.020), sphinganine (p<0.001), S1P (p<0.001), C16DHC (p<0.01) and C20Cer (p<0.001) and C18Cer (p<0.001) level variations associated to the virologic course of HBV infection. In addition, serum S1P- und dhS1P-levels are elevated in HBV genotype D infected patients. Further studies are certainly required to elucidate sphingolipids as potential novel predictors of the natural course of HBeAg-negative HBV infection.

## Supporting information

S1 Fig Patients’ selection. (TIF)

S2 Fig Course of liver stiffness. Our patients show no significant progression or regression of liver stiffness in yearly transient elastography measurements. Bars depict mean +/- standard mean error.
(TIF)

S3 Fig **Course of (A) sphingosine-1-phosphate, (B) C24Cer, (C) C16Cer, (D) C14Cer, (E) C24:1Cer, (F) C18DHC, (G) C24DHC and (H) C24:1DHC in patients with no virologic events from baseline (BL) over a follow-up (FU) period of four years (1–4).** Here depicted are all sphingolipid parameters without significant changes over time. Bars depict mean +/- standard mean error.
(TIF)

S4 Fig Kruskal-Wallis analyses at baseline between patients with different upcoming virologic events (VE). There are no significant differences in the here listed (A-K) sphingolipid (SL) parameters. In patients with upcoming hepatitis B antigen (HBsAg) + hepatitis B antibody (HBsAb) status, concentrations of (H) C16DHC and (I) C18DHC could not be quantified. Bars depict mean +/- standard mean error.
(TIF)

S5 Fig **Alterations of (A) C16DHC, (B) C18DHC, (C) C24DHC and (D) C24:1DHC in all patients with hepatitis B (HBV) reactivation. Sphingolipids are compared at last visit before and next visit after HBV reactivation.** There are no significant dynamics in all shown dihydroceramides (A-D). Statistically significant differences are indicated by asterisks. "*"p<0.05, "**"p<0.01, "***"p<0.001. Bars depict mean +/- standard mean error.
(TIF)

S6 Fig **Alterations of (A) sphinosine, (B) sphinganine, (C) sphingosine-1-phosphate and (D) sphinganine-1-phosphate in all patients with hepatitis B (HBV) reactivation.** Sphingolipids are compared at last visit before and next visit after HBV reactivation. There are no significant dynamics in sphingosine and sphinganine (A,B) or their phosphate derivates (C,D). Statistically significant differences are indicated by asterisks. "*"p<0.05, "**"p<0.01, "***"p<0.001. Bars depict mean +/- standard mean error.
(TIF)

S1 Table Hospitals and affiliated centres included in this multicentre trial. (DOCX)

## Abbreviations

HBV: hepatitis B virus
HCC: hepatocellular carcinoma
SL: sphingolipid
HCV: hepatitis C virus
HBeAb: hepatitis B envelope antibody
HBsAg: hepatitis B surface antigen
HBeAg: hepatitis B envelope antigen
HBsAb: hepatitis B surface antibody
BMI: body mass index
TE: transient elastography
PEG-INF: pegylated interferon
Chol: cholesterol
TG: triglyceride
AFP: alpha fetoprotein
VL: viral load
DHC: dihydroceramide
LDL: low density lipoprotein
HDL: high density lipoprotein
ALT: alanine transferase
AST: aspartate transferase
