# Supplementary figures and images for: Serum sphingolipid levels associate with upcoming virologic events and HBV genotype D in a cohort of patients with HBeAg-negative HBV infection
Source: PLoS One. 2018 Nov 15;13(11):e0207293. doi: 10.1371/journal.pone.0207293 (PMC6237377; doi:10.1371/journal.pone.0207293)

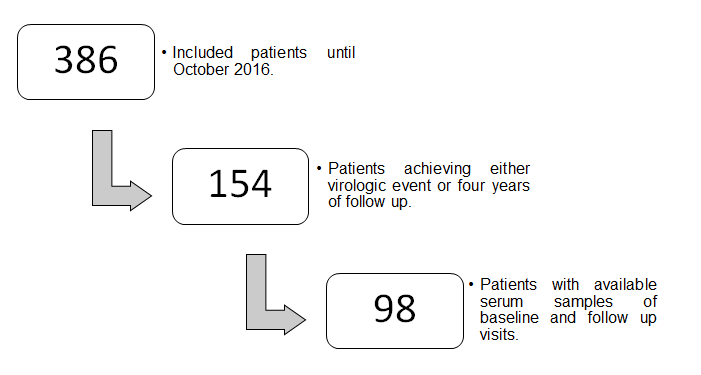

Supplement: S1 Fig — (TIF) [file pone.0207293.s001.tif]

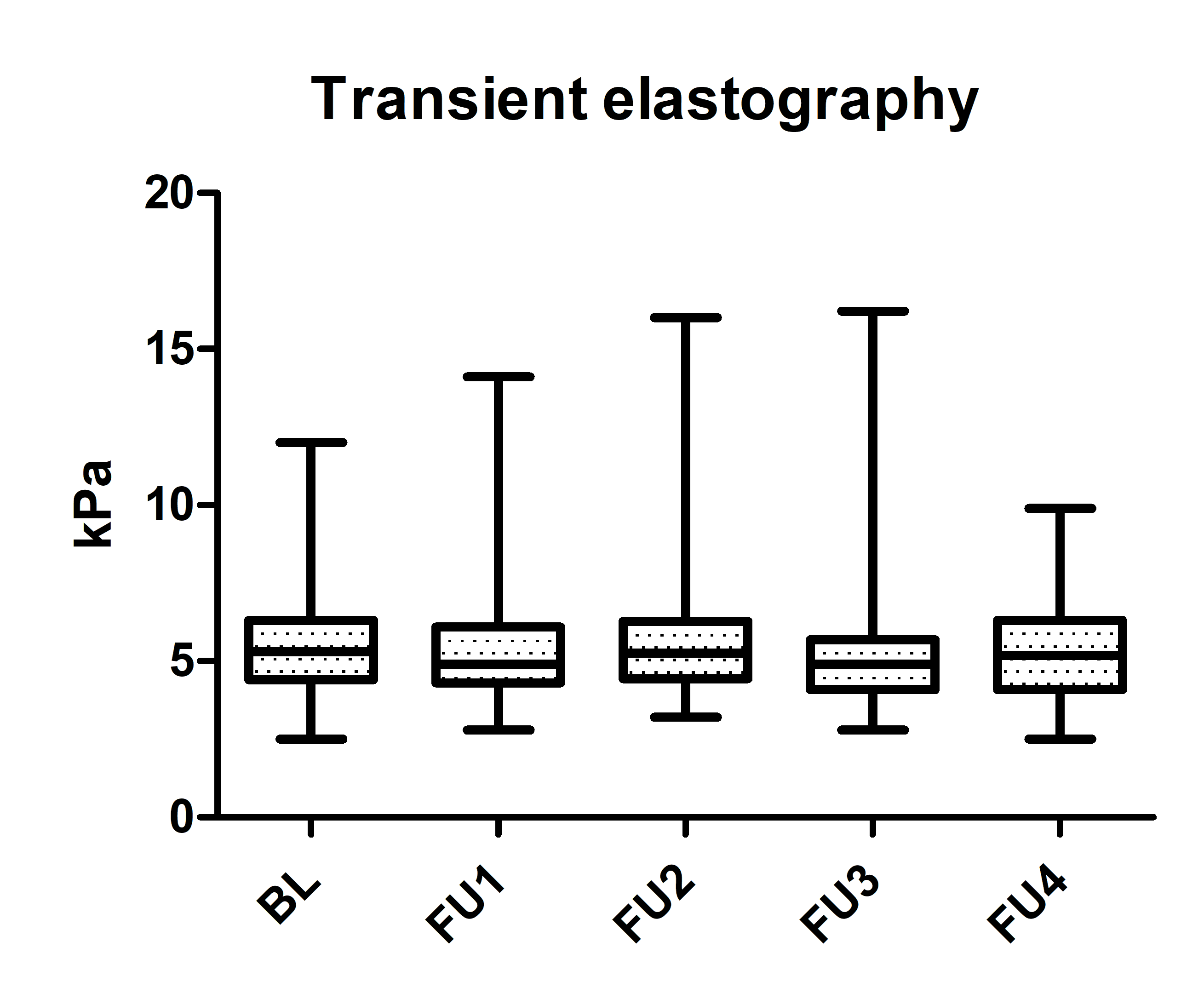

Supplement: S2 Fig — Our patients show no significant progression or regression of liver stiffness in yearly transient elastography measurements. Bars depict mean +/- standard mean error. (TIF) [file pone.0207293.s002.tif]

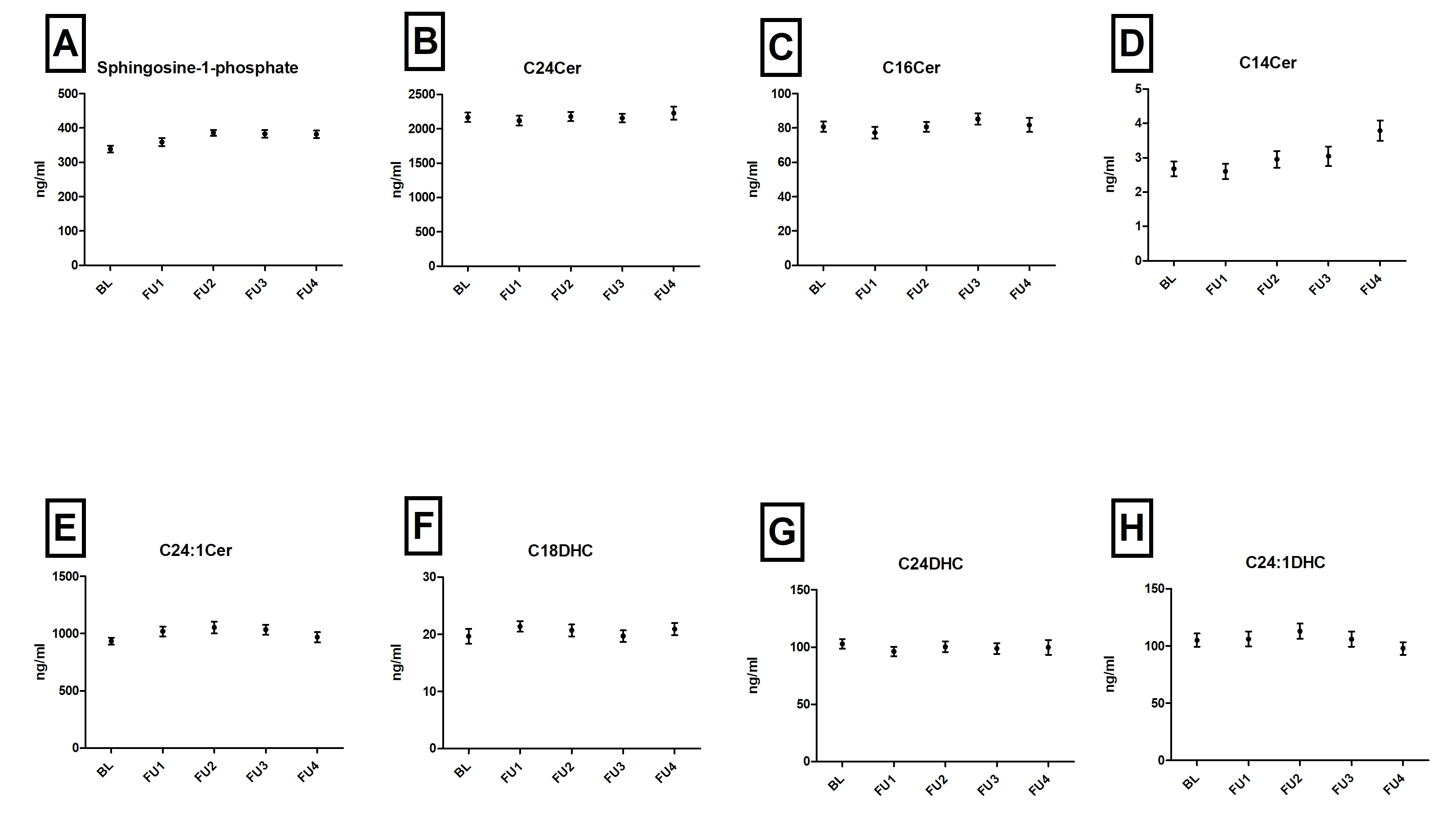

Supplement: S3 Fig — Course of (A) sphingosine-1-phosphate, (B) C24Cer, (C) C16Cer, (D) C14Cer, (E) C24:1Cer, (F) C18DHC, (G) C24DHC and (H) C24:1DHC in patients with no virologic events from baseline (BL) over a follow-up (FU) period of four years (1–4). Here depicted are all sphingolipid parameters without significant changes over time. Bars depict mean +/- standard mean error. (TIF) [file pone.0207293.s003.tif]

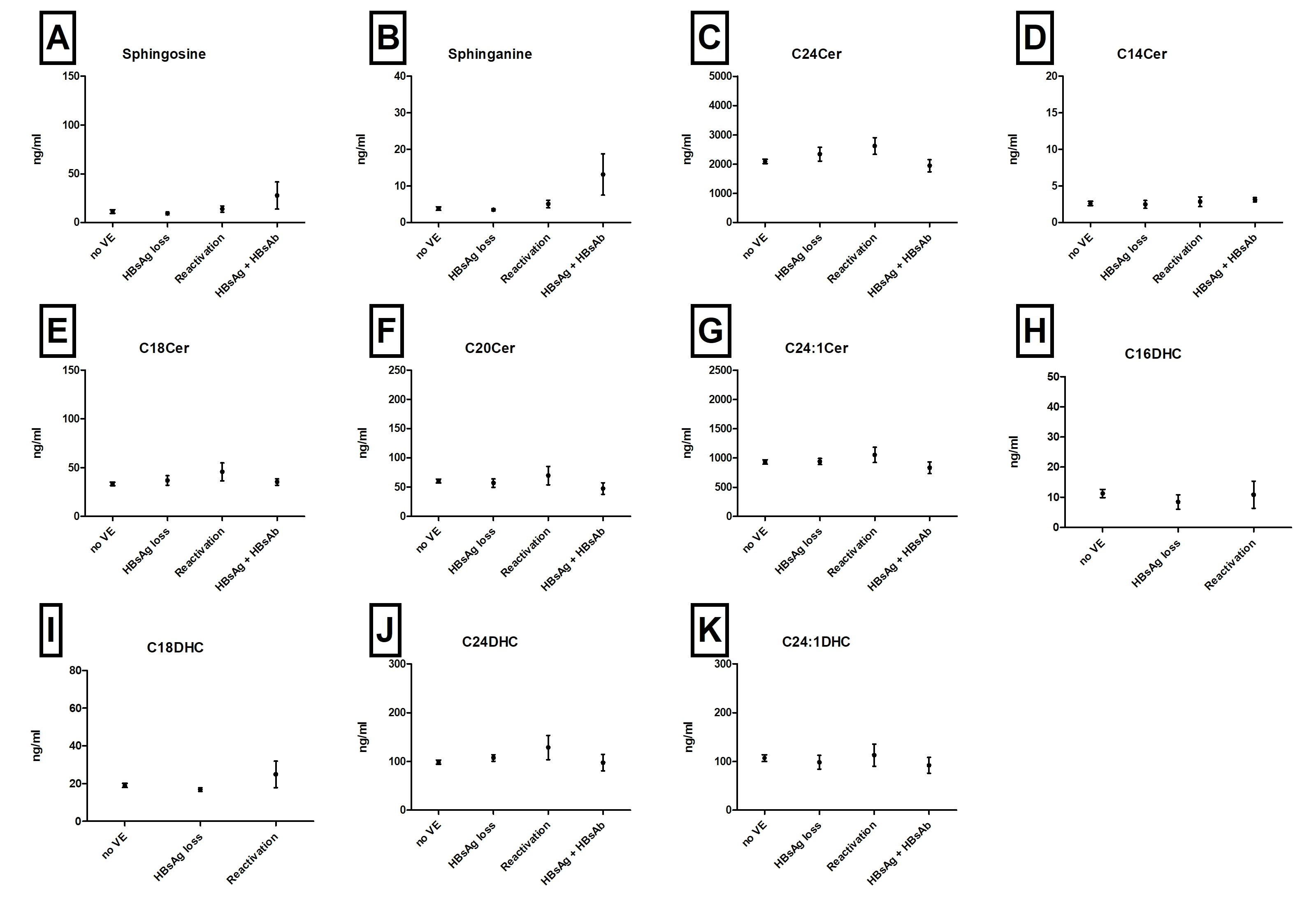

Supplement: S4 Fig — There are no significant differences in the here listed (A-K) sphingolipid (SL) parameters. In patients with upcoming hepatitis B antigen (HBsAg) + hepatitis B antibody (HBsAb) status, concentrations of (H) C16DHC and (I) C18DHC could not be quantified. Bars depict mean +/- standard mean error. (TIF) [file pone.0207293.s004.tif]

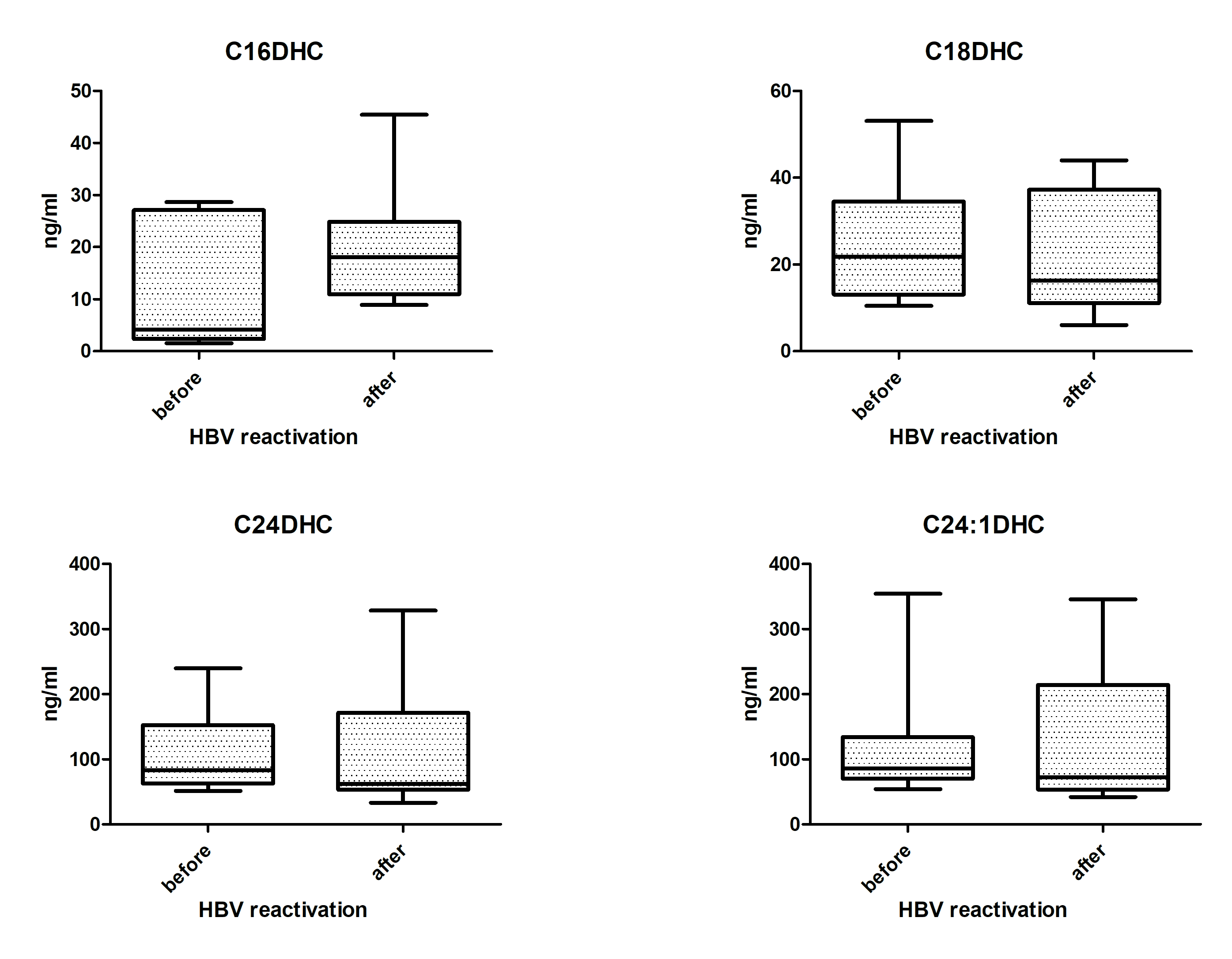

Supplement: S5 Fig — Alterations of (A) C16DHC, (B) C18DHC, (C) C24DHC and (D) C24:1DHC in all patients with hepatitis B (HBV) reactivation. Sphingolipids are compared at last visit before and next visit after HBV reactivation. There are no significant dynamics in all shown dihydroceramides (A-D). Statistically significant differences are indicated by asterisks. "*"p<0.05, "**"p<0.01, "***"p<0.001. Bars depict mean +/- standard mean error. (TIF) [file pone.0207293.s005.tif]

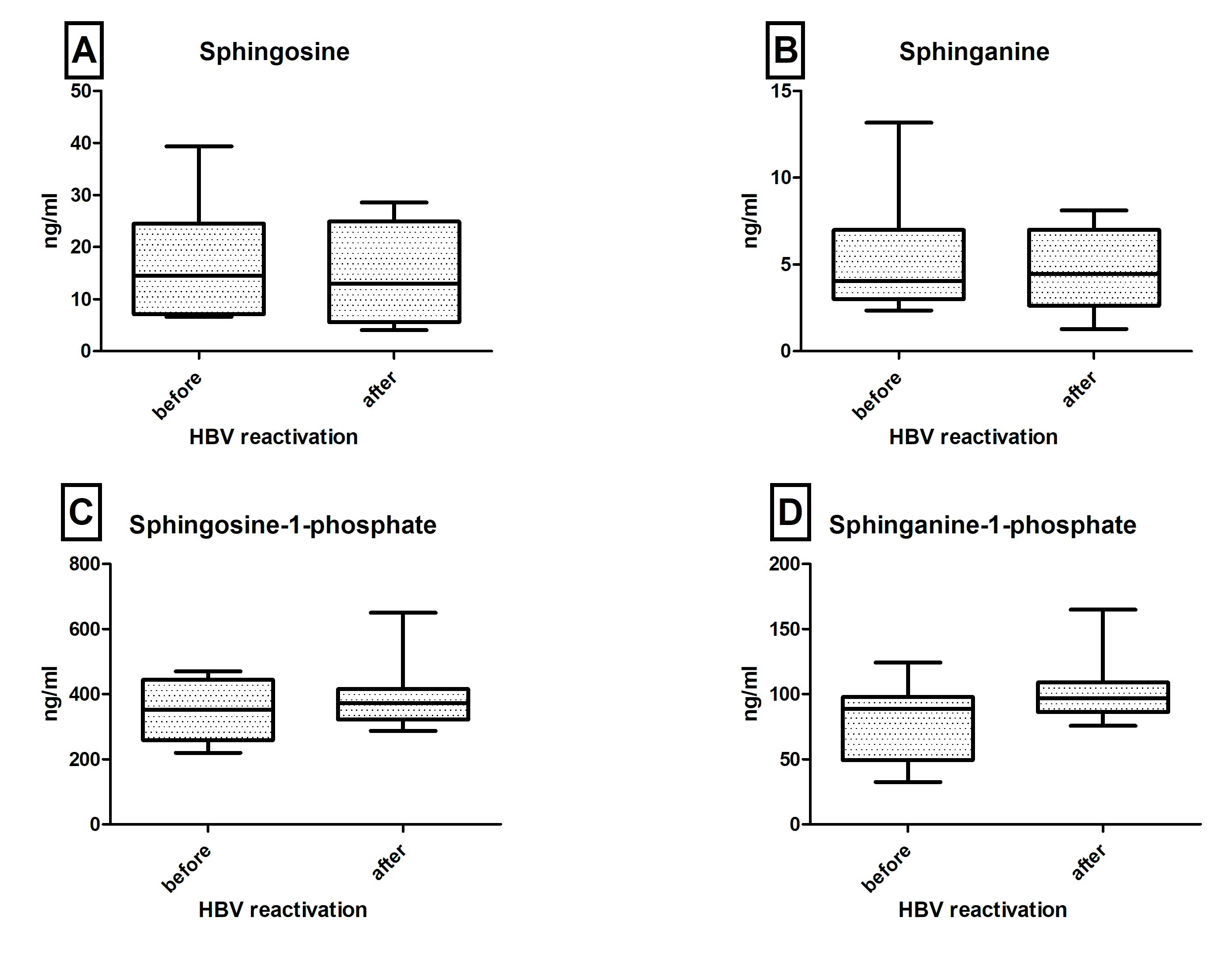

Supplement: S6 Fig — Alterations of (A) sphinosine, (B) sphinganine, (C) sphingosine-1-phosphate and (D) sphinganine-1-phosphate in all patients with hepatitis B (HBV) reactivation. Sphingolipids are compared at last visit before and next visit after HBV reactivation. There are no significant dynamics in sphingosine and sphinganine (A,B) or their phosphate derivates (C,D). Statistically significant differences are indicated by asterisks. "*"p<0.05, "**"p<0.01, "***"p<0.001. Bars depict mean +/- standard mean error. (TIF) [file pone.0207293.s006.tif]
