# Supplementary material for: Serum sphingolipid levels associate with upcoming virologic events and HBV genotype D in a cohort of patients with HBeAg-negative HBV infection
Source: PLoS One. 2018 Nov 15;13(11):e0207293. doi: 10.1371/journal.pone.0207293 (PMC6237377; doi:10.1371/journal.pone.0207293)
Supplement: S1 Table — (DOCX) [file pone.0207293.s007.docx]

**S1 Table. Hospitals and affiliated centres included in this multicentre trial.** ClinicalTrials.gov Identifier: NCT01090531

| **No.** | **Centre** | **Place** |
| --- | --- | --- |
| 001 | Universitätsklinikum Frankfurt am Main | 60590 Frankfurt |
| 002 | Praxis, Gastroenterologie | 44623 Herne |
| 003 | Medizinische Hochschule Hannover | 30625 Hannover |
| 004 | Praxis, Innere Medizin | 40237 Düsseldorf |
| 005 | Ifi-Institut GmbH | 20099 Hamburg |
| 006 | Universitätsklinikum Leipzig | 04103 Leipzig |
| 007 | Praxis, Innere Medizin | 10777 Berlin |
| 008 | Universitätsklinikum Mainz | 55101 Mainz |
| 009 | Universitätsklinikum Würzburg | 97080 Würzburg |
| 010 | Praxis, Gastroenterologie | 24146 Kiel-Elmschenhagen |
| 011 | Klinikum, Nancy | 54511 Vandoevre-Les Cedex |
| 012 | Gastro-Data Wiesbaden | 65189 Wiesbaden |
